# Development of Ulvan-Containing Liposomes as Antibacterial Drug Delivery Platforms

**DOI:** 10.3390/jfb13040186

**Published:** 2022-10-13

**Authors:** Leto-Aikaterini Tziveleka, Natassa Pippa, Efstathia Ioannou, Costas Demetzos, Vassilios Roussis

**Affiliations:** 1 Section of Pharmacognosy and Chemistry of Natural Products, Department of Pharmacy, National and Kapodistrian University of Athens, Panepistimiopolis Zografou, 15771 Athens, Greece; 2 Section of Pharmaceutical Technology, Department of Pharmacy, National and Kapodistrian University of Athens, Panepistimiopolis Zografou, 15771 Athens, Greece

**Keywords:** ulvan, liposomes, drug delivery, nanocarrier, antibacterial activity

## Abstract

Liposomes, due to their safety profile and targeting ability, are among the most studied nanocarriers as antimicrobial delivery systems. However, due to lack of stability and the non-specific interaction of liposomes with cells and proteins, their use is relatively limited. Aiming to overcome these drawbacks, it was envisaged that incorporation of ulvan, a bioactive marine sulfated polysaccharide isolated from green algae, in liposomes could improve their physicochemical properties and overall stability. Thus, we initially studied the interactions of ulvan with neutral, negatively, and positively charged lipids using Differential Scanning Calorimetry and subsequently, based on the obtained results, we prepared the respective ulvan–containing neutral and charged liposomes, where ulvan interacts with both lipid chains and polar groups in the liposomal bilayer. In a further step, we entrapped in the liposomes fusidic acid, used as a model antibacterial drug, and proceeded with the evaluation of their antibacterial activity against *Staphylococcus aureus*. The physicochemical properties (size and ζ-potential), stability, morphology, and entrapment efficiency of the prepared liposomal formulations were determined.

## 1. Introduction

The use of nanopharmaceuticals has been recently recognized as a promising strategy for the successful delivery of antibiotics [1]. Liposomes, due to their safety profile and targeting ability, are among the most studied nanocarriers as antimicrobial delivery systems [2,3,4,5]. The preparation of liposomes with desired characteristics is accomplished by manipulating diverse liposomal physical properties, including bilayer composition and membrane fluidity, size, surface charge, and coating. The liposomal lipidic bilayer’s structure resembles that of cell membranes, therefore enabling fusion with bacterial membranes [6]. Consequently, antibiotics encapsulated within the liposomes can be released to the cell membrane or the interior of a bacterial cell [7], resulting in high drug concentrations locally [6].

However, lack of stability and the non-specific interaction of liposomes with cells and proteins, leading to drug accumulation in non-target tissues, limit their use. These drawbacks can be overcome via surface modification of the liposomes, with polyethylene glycol (PEG) coatings being the most widely used strategy [8].

Polysaccharides, exhibiting varying physicochemical properties according to their structures and functional groups, have been considered as an alternative to PEG coatings [9]. In addition to their well-acknowledged biocompatibility and biodegradability, considered as indispensable properties for polymers used as biomaterials [10], they also display important biological activities, such as antiviral, antibacterial, antitumoral, anti-inflammatory, and antioxidant [10,11,12].

Polysaccharides are extensively expressed in cell membranes and involved in cell surface properties, including cellular recognition and adhesion [13]. Taking into consideration the role of polysaccharides in biological or biomimetic activities, the design of polysaccharide–containing carriers appears appealing [14]. In this context, dextran, pullulan, and glycolipids have been used as coatings to decrease the uptake of liposomes by the mononuclear phagocyte system [15,16]. Furthermore, hyaluronic acid has been found to confer bioadhesive properties to liposomes for local drug depot [17], while liposome–hyaluronic acid hybrid nanoparticles have been developed for intranasal vaccination [18]. Moreover, functionalized dextran was shown to enable targeting of human endothelial cells [19] and vascular smooth muscle cells [20], whereas pullulan was useful for oral immunization [21]. Chitosan–containing liposomes have been used for controlled drug release to the lungs by inhalation [22], to the intestine by oral administration [23], or for the topical therapy of vaginal infections [24,25], as well as for the treatment of antibiotic-resistant bacterial infections [26]. Additionally, chitosan and dextran sulfate-coated liposomes have been prepared to enable the spatial control of calcium phosphate formation over the capsule surface for biomineralization [27].

Ulvan, a water soluble and complex anionic sulfated polysaccharide, represents the major constituent of the cell walls of green seaweeds of the order Ulvales (Chlorophyta) [28]. The physical properties and pharmacological activity of ulvan have been systematically investigated [29,30,31] and a wide range of biological activities have been reported [32,33,34,35]. So far, a number of ulvan-based scaffolds [36,37,38,39,40,41,42], polyelectrolyte complex materials [43,44,45], 2D crosslinked membranes [46], and nanofibers [47,48,49] have been prepared.

In the current study, aiming to develop ulvan–containing liposomes as effective antibacterial drug delivery systems, the interactions between neutral, negatively, and positively charged membrane lipids and ulvan were initially investigated with Differential Scanning Calorimetry (DSC). Based on the results obtained, plain and ulvan–containing liposomes were prepared, along with the respective formulations encapsulating fusidic acid (FA) as a model antibacterial drug. The size, charge, and entrapment efficiency of all vesicles were determined by Dynamic Light Scattering (DLS), measurement of ζ-potential, and UV-Vis spectroscopy, respectively. The morphology of the ulvan–containing liposomes was examined by Scanning Electron Microscopy (SEM), while the antibacterial activity of both the plain and modified liposomes was evaluated against the Gram-positive bacterium *Staphylococcus aureus*.

## 2. Materials and Methods

### 2.1. Materials

Specimens of the green alga *Ulva rigida*, collected in Chalcis in the island of Euboea, Greece, were used for the isolation of ulvan. The isolation, chemical analysis, and deproteinization of ulvan were carried out as previously described [45].

### 2.2. Chemicals

**Scheme 1:**
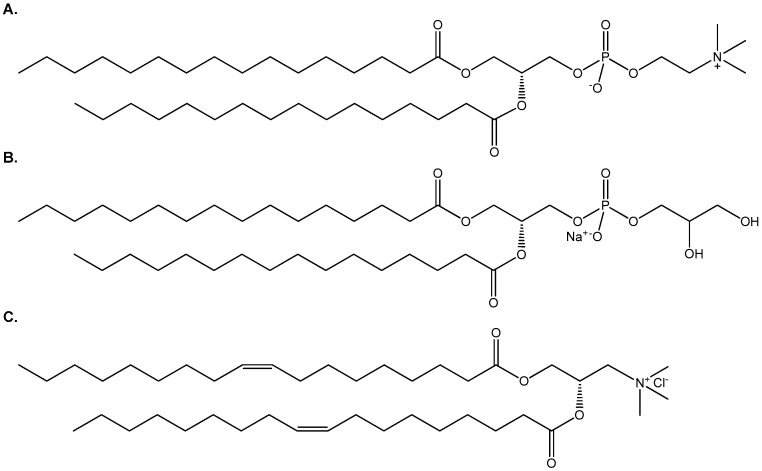
The chemical structures of (**A**) DPPC, (**B**) DPPG and (**C**) DOTAP.

1,2-Dipalmitoyl-*sn*-glycero-3-phosphocholine (DPPC), 1,2-dipalmitoyl-*sn*-glycero- 3-phospho-(1′-*rac*-glycerol) (DPPG) and 1,2-dipalmitoyl-3-trimethylammonium-propane (DOTAP) were purchased from Avanti Polar Lipids Inc. (Albaster, AL, USA). The chemical structures of the lipids are presented in Scheme 1. HPLC grade CHCl_3_ and MeOH were purchased from LabScan (Dublin, Ireland). Tris(hydroxymethyl) aminomethane and CaCl_2_·2H_2_O were obtained from Mallinckrodt (Dublin, Ireland), EDTA from Serva (Heidelberg, Germany), MgCl_2_·6H_2_O and FA from Merck (Kenilworth, NJ, USA), Mueller-Hinton broth from OXOID (Hampshire, UK), Mueller-Hinton 2 Agar from BioMérieux (Marcy-l’Étoile, France), while sodium dodecyl sulfate (SDS) and 3-(4,5-dimethylthiazol-2-yl)-2,5-diphenyltetrazolium bromide (MTT) were purchased from Sigma (Darmstadt, Germany).

### 2.3. Preparation of Ulvan–Containing Liposomes

**Scheme 2:**
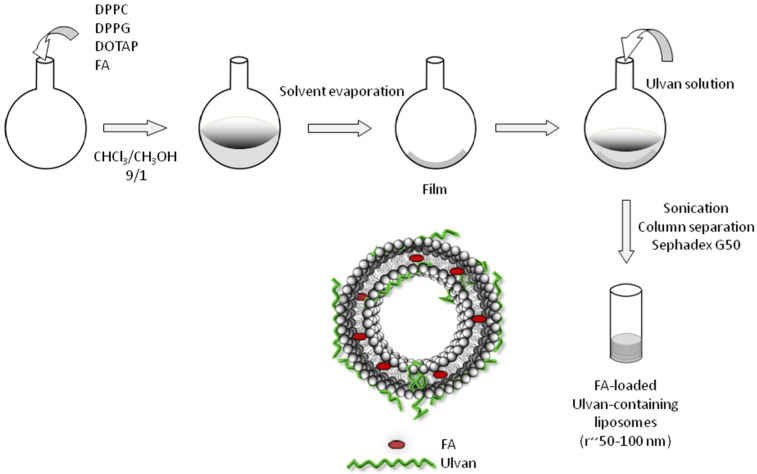
Schematic representation of the preparation protocol of ulvan-containing liposomes.

Different liposomal formulations were prepared using the thin-film hydration method. Briefly, appropriate amounts of phospholipid mixtures composed of DPPC, DPPC:DPPG (9:1 molar ratio) and DPPC:DOTAP (9:1 molar ratio) were dissolved in CHCl_3_/MeOH solution (9:1, *v/v*). The solvents were evaporated at 40 °C in a rotary evaporator (Rotavapor R-200, Buchi, Switzerland) for the formation of lipid films. Each film was maintained under vacuum overnight and subsequently hydrated with ulvan solution in HPLC grade water or in Phosphate Buffered Saline (PBS) by slow stirring for 1 h in a water bath at temperatures above the phase transition of lipids (41 °C for DPPC and DPPC:DPPG and 40 °C for DPPC:DOTAP). The resultant multilamellar vesicles (MLVs) were subjected to two 3-min sonication cycles (amplitude b70, cycle 0.7) interrupted by a 3-min resting period, using a probe sonicator (UP 200S, dr. Hielsher GmbH, Berlin, Germany). The resultant vesicles (LDPPC-UL, LDPPG-UL, LDOTAP-UL) were allowed to anneal for 30 min. The as-prepared vesicles were separated on Sephadex G50 columns. The preparation protocol of ulvan–containing liposomes is presented in Scheme 2.

### 2.4. Preparation of FA-Loaded Liposomes

FA-loaded liposomes, both plain and ulvan–containing, were prepared by the thin-film hydration technique, according to the procedure described above. Accurately weighed amounts of the drug (FA) and phospholipids were dissolved in a solvent mixture (CHCl_3_/MeOH, 9:1 *v/v*). The amount of drug (2% *w/w*) was kept constant. The obtained dry thin lipid film was then hydrated for 1 h using ulvan solution (1 mg/mL) in HPLC grade water at temperatures higher than the T_m_ of the lipids. Afterwards, the preparation procedure described for the ulvan–containing liposomes (Section 2.3) was followed.

### 2.5. Scanning Electron Microscopy

For SEM analyses, a drop of the selected samples was placed on the sample stab for 24 h at room temperature until the samples were completely dried and subsequently coated with a conductive layer of sputtered gold. The SEM images were recorded in a PhenomWorld desktop scanning electron microscope (Thermo Fischer Scientific, Waltham, MA, USA) with tungsten filament (10 kV) and charge reduction sample holder.

### 2.6. FTIR Spectroscopy

FTIR spectra of the lyophilized ulvan–containing liposomes, as well as of pure DPPC and ulvan, were recorded on an FTIR Bruker Alpha II (Billerica, MA, USA) using the attenuated total reflection method. All spectra were recorded at room temperature and averaged from 24 scans in the range of 400–4000 cm^−1^ with a resolution of 4 cm^−1^.

### 2.7. Particle Size Analysis

Dynamic and Electrophoretic Light Scattering (DLS and ELS) techniques were employed for the physicochemical characterization of ulvan and the prepared plain and ulvan–containing liposomal systems. The mean hydrodynamic diameter (D*h*), polydispersity index (PDI) and ζ-potential of the particles were used for the characterization of the liposomal systems immediately after preparation (t = 0 days), as well as for monitoring their physical stability over time (t = 28 days). Measurements were performed at a detection angle of 90° and at 25 °C in a photon correlation spectrometer (Zetasizer 3000 HSA, Malvern Pananalytical Ltd., Malvern, UK) and analyzed by the CONTIN method (Malvern software, UK). For each system, three sets of ten light scattering measurements were collected and the results were averaged.

### 2.8. Zeta Potential Determination

The ζ-potential of the as prepared particles was measured using Zetasizer 3000 HSA (Malvern Panalytical Ltd., Malvern, UK). Specifically, 200 μL of dispersion was diluted in 3 mL of HPLC grade water, and ζ-potential was measured at room temperature at 633 nm. The ζ-potential values were calculated from electrophoretic mobilities, *μ_Ε_*, by using the Henry correction of the Smoluchowski equation:

$$\zeta =\frac{3\mu_{Ε}n}{2\varepsilon_{0\;}\varepsilon_{r}}\frac{1}{f\left(\kappa a\right)}$$

where *ε_0_* is the permittivity of the vacuum, *ε_r_* is the relative permittivity, *α* is the particle radius, *κ* is the Debye length, and *n* is the viscosity of water. Experiments were run in triplicate.

### 2.9. Differential Scanning Calorimetry

DSC experiments were performed on an 822e Mettler-Toledo (Schwerzenbach, Switzerland) calorimeter calibrated with pure indium (T_m_ = 156.6 °C) and water. Sealed aluminum 40 μL crucibles were used as sample holders. DPPC, DPPC:DPPG (9:1 molar ratio), and DPPC:DOTAP (9:1 molar ratio) lipids were dissolved in CHCl_3_, and the solvent was removed by slow evaporation, while residual solvents were removed under vacuum overnight. The dried material was weighted into the aluminum crucibles and lipid bilayers were prepared by hydrating with ulvan dispersions in different concentrations (i.e., 1, 2.5, 5, 7.5, and 10 mg/mL). The DPPC, DPPC:DPPG (9:1 molar ratio) and DPPC:DOTAP (9:1 molar ratio) lipid bilayers were hydrated in ulvan dispersion with an overall known lipid content. An empty aluminum crucible was used as reference. Prior to measurements, the crucibles were heated at a temperature that exceeds the transition of DPPC (41.7 °C) to ensure equilibration. Three heating–cooling cycles were performed at the temperature range from 10 to 60 °C with a rate of 2 and 20 °C/min for heating and cooling, respectively. Thermodynamic evaluation was performed during the second cooling and heating cycle. All samples were scanned until identical curves were obtained. Errors were evaluated on the basis of at least three replicates. Enthalpy changes and characteristic transition temperature were calculated with 822e Mettler-Toledo STARe software.

### 2.10. Determination of Entrapment Efficiency

To remove unentrapped FA from the plain and ulvan–containing liposomes, the vesicles were separated on Sephadex G50 columns. After complete saturation of the columns, FA-loaded vesicular dispersion (0.5 mL) was placed in the column. The column was rinsed using water and the drug-entrapped liposomes were pooled. Thereafter, the eluted dispersion was either lyophilized (CoolSafe, ScanVac) for 24 h, weighed and analyzed for its entrapment efficiency, or used as-is for the antibacterial activity evaluation [50].

The lyophilized samples were dissolved in CHCl_3_/MeOH (1:1) and centrifuged. The supernatant was collected, and the liposomally entrapped FA was quantified using UV–Vis spectroscopy by monitoring on a Perkin Elmer Lambda 40 spectrophotometer the absorbance of FA at 228 nm after subtracting the spectra of the employed lipids at the respective concentration. Calculations were based on the standard calibration curve of FA solution prepared in CHCl_3_/MeOH (1:1) using concentrations in the range of 6 to 166 μg/mL (R^2^ = 0.9982).

### 2.11. Evaluation of Antibacterial Activity

The determination of minimum inhibitory concentration (MIC) values was conducted as previously described [51]. Briefly, *S. aureus* (strain ATCC 25923) cells were initially cultured on nutrient agar and incubated for 24 h at 37 °C. Single colony cells were grown in MH medium at 37 °C in an orbit shaker under constant agitation (180 rpm). Bacterial inoculum equivalent to the 0.5 McFarland turbidity standard was prepared in normal saline and diluted to a final density of 5 × 10^5^ cfu/mL. MHB supplemented with 10 mg/L Mg^2+^ and 20 mg/L Ca^2+^ was dispensed into the wells of 96-well microtiter plates. The liposomal solutions were added to the first well of each row and were serially diluted across the row. The inoculum was added to the wells of each row, and the microtiter plates were incubated for 18 h at 37 °C. Wells free of test samples served for growth control, while MHB and inoculum-free wells served as sterility control. The lowest concentration at which no bacterial growth was observed was recorded as the MIC. The observation was confirmed by the addition of a 5 mg/mL methanolic solution of MTT (20 μL/well) and further incubation for 20 min at 37 °C. Bacterial growth was indicated by color alteration from yellow to dark blue. FA was employed as positive control for *S. aureus*. Ulvan solutions and drug-free liposomal solutions were also tested. All samples were tested in triplicate.

## 3. Results and Discussion

### 3.1. Thermodynamic Evaluation of Lipid-Ulvan Interactions

As a first step, we studied the interactions of different concentrations of ulvan with DPPC, DPPC:DPPG, and DPPC:DOTAP lipid bilayers. DPPC lipid bilayers are zwitterionic, while DPPC:DDPG and DPPC:DOTAP lipid bilayers are anionic and cationic, respectively, due to the charged lipids they contain (Scheme 1). The lipids’ molar ratio in the mixed bilayers was 9:1 on the basis of previously published studies on model membranes [52,53,54].

**Figure 1:**
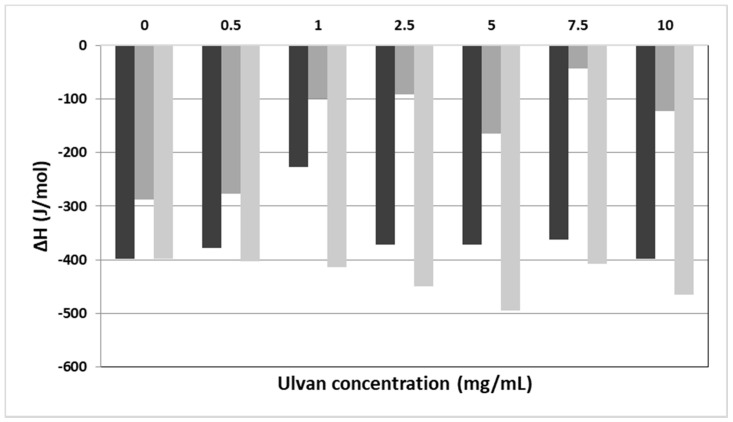
The Δ*H* values vs. the concentration of ulvan (mg/mL) employed for the hydration of DPPC (black bars), DPPC:DPPG (9:1 molar ratio) (dark grey bars), and DPPC:DOTAP (9:1 molar ratio) (light grey bars) membranes.

In Table 1 and Figure 1, the calorimetric values of the examined systems are presented. The selected values included T_onset_: temperature at which the thermal event starts, T: temperature at which heat capacity (Δ*Cp*) at constant pressure is maximum, ΔT_1/2_: half width at half peak height of the transition, and Δ*H*: transition enthalpy normalized per mol of lipid bilayer system. Hence, we studied how these values were altered in the presence of increasing ulvan concentrations.

Concerning the pure DPPC bilayers, two main thermal events are observed. The pretransition peak (T_s_) referring to the lamellar gel to rippled phase transition of lipids is quantitatively synonymous to the mobility of head groups of lipids. On the other hand, the main transition (T_m_) temperature is quantitatively synonymous to the mobility of alkyl chains and is referring to the rippled to liquid crystalline phase. The addition of a guest molecule (i.e., active pharmaceutical ingredient, polymer, protein/peptide, etc.) in the lipid bilayer causes alterations of its thermodynamic characteristics. The main transition temperature is a unique property of each lipid. For pure DPPC lipids, the T_m_ is equal to 41 °C, very close to the observed value (41.9 °C) (Table 1) [52,53].

Upon addition of different concentrations of ulvan solution in the DPPC bilayers no effect on the thermal event of the main transition temperature initiation (T_onset,m_ values, Table 1) was observed. The T_m_ increased slightly, with the maximum increase being 1.6 °C at the highest concentration of ulvan used (T_m_ values, Table 1). The enthalpy of melting remained more or less unaffected in the presence of ulvan. The only exception observed in the Δ*H*_m_ values was at an ulvan concentration of 1 mg/mL, where the enthalpy decreased in absolute values to almost half as compared to pure DPPC bilayer (Δ*H*_m_ values, Table 1). The ΔT_1/2_._m_ values increased upon addition of the ulvan solution as compared with those of the pure lipids, pointing to differences in the cooperativity between the alkyl chains (ΔT_1/2_._m_, Table 1). This phenomenon is strongly dependent on the mobility of head groups. According to the calorimetric values of pretransition, although pretransition temperature (T_s_) did not change significantly, a very significant reduction in absolute values in enthalpy (Δ*H*_s_) was observed when ulvan concentration increased. This indicates that more polymer chains interact with the polar head groups by modifying their mobility and causing reduction of the pretransition enthalpy.

Following the initial investigation on the interaction of ulvan with DPPC lipid bilayers, the most ordinary cellular membrane model, we extended our investigation on negatively charged lipid bilayers, with the negative charge stemming from the DPPG lipid. Therefore, we used mixed DPPC:DPPG lipid bilayers at a 9:1 molar ratio, with both DPPG and DPPC lipids sharing the same T_m_ of 43.1 °C [52,53]. The main difference between DPPC and DPPC:DPPG lipid bilayers is the complete abolishment of pretransition peak upon ulvan addition (Table 1). The repulsion between the negatively charged head groups of DPPG and the negatively charged ulvan chains caused the increase of the mobility of the head groups (Table 1, absence of the values in the pretransition thermal phenomenon), while the T_m_ decreased marginally. On the other hand, upon addition of ulvan, there was a strong reduction of the enthalpy of the main transition event but not in parallel to the increase of ulvan concentration. According to the DSC data, the ulvan chains are not incorporated deep into the DPPC:DPPG lipid bilayers due to the negatively charged membranes. Generally, a profound incorporation of polymeric chains or other molecules into lipid bilayers causes significant reduction of the absolute values of Δ*H*_m_ [52,53]. The reduction of Δ*H*_m_ values (Figure 1, Table 1) is due to the fluidity increase of the lipid bilayer stemming from the increased mobility of the head groups. Another possible explanation for this behavior is the increase in the distance between the alkyl chains of the phospholipids due to the stereochemical repulsion generated by the ulvan chains. The van der Waals interactions are weakened, so less energy is required for the transition to the liquid crystalline phase.

The effect of the cationic lipid DOTAP on both the thermotropic phase behavior and the structural organization of aqueous DPPC dispersions using DSC and DLS measurements have already been reported in the literature [55]. For comparison purposes, we selected the same molar ratio (9:1) as in the DPPC:DPPG mixture for studying the interactions of ulvan with the DPPC:DOTAP lipid bilayer. The T_m_ of DOTAP is −8 °C. As in the case of DPPC:DPPG bilayer, marginal changes of the T_onset.m_ and T_m_ values and abolishment of the pretransition was observed. On the contrary, we observed a significant increase of the Δ*H*_m_ absolute values (Figure 1), denoting the enhancement of the energy required for the transition from rippled gel to the lamellar liquid crystalline phase. The strong attractive interactions between the COO^−^ groups of ulvan and the NH_4_^+^ groups of DOTAP caused the incorporation of the ulvan chains into the lipid bilayers. Due to this phenomenon, the decrease of the distance between the alkyl chains increased the van der Waals interactions and consequently the required enthalpy for the melting of ulvan-grafted mixed lipid bilayers.

### 3.2. Preparation and Characterization of Ulvan–Containing Liposomes

To study the formation of ulvan–containing liposomes, an ulvan concentration of 1 mg/mL was selected based on the above presented DSC results (Table 1), where the highest cooperative interactions in all lipid membrane models were achieved in these ulvan to lipid ratios. Ulvan solutions in both HPLC grade water and PBS were employed since the dispersion medium influences the size and the ζ-potential of the self-assembled particles. Additionally, neutral, negatively, and positively charged liposomes using different lipids in HPLC grade water were also prepared, so as to compare their physicochemical characteristics to the ones of the respective ulvan–containing liposomes.

**Figure 2:**
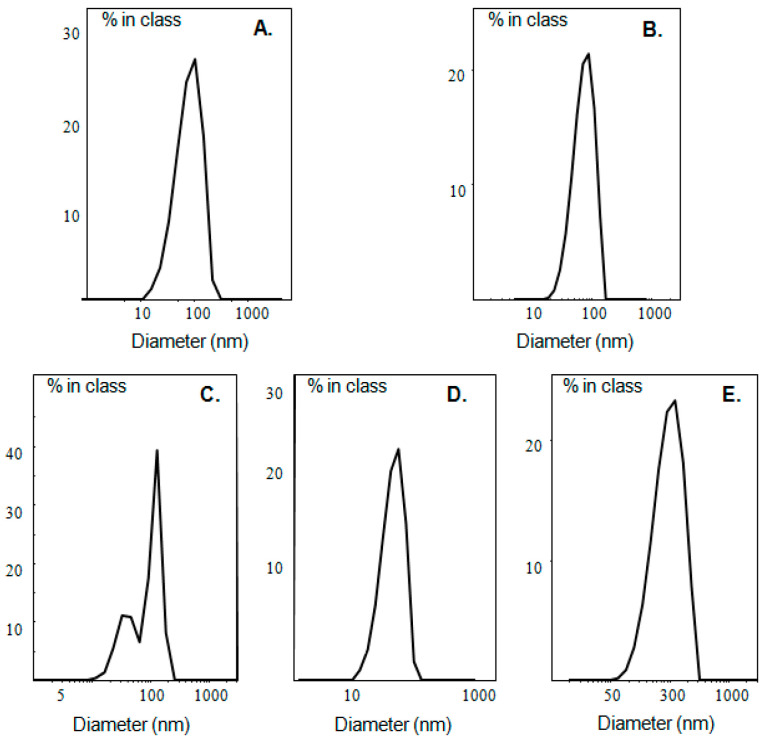
Size distribution of (**A**) LDPPG-UL and (**B**) LDOTAP-UL in HPLC grade water, as well as (**C**) LDPPC-UL, (**D**) LDPPG-UL, and (**E**) LDOTAP-UL in PBS.

The physicochemical characteristics (size, size distribution, ζ-potential) of the prepared liposomes, as well as of pure ulvan are presented in Table 2 and Figure 2. Ulvan particles formed in HPLC grade water exhibited size around 580 nm and negative ζ-potential, while when ulvan was dispersed in PBS, both size and ζ-potential decreased (Table 2). In both media, the size distribution was heterogeneous with the recorded curves being broad, probably due to ulvan’s polyelectrolyte nature and the low concentration employed, resulting in limited steric repulsion effects of the polyelectrolyte chains (Figure 2, Table 2).

The colloidal dispersion for all ulvan–containing liposomes was equal to 30 mg/mL, and 33 μg ulvan were added per 1 mg of liposome suspension. In the case of neutral DPPC lipid films, hydration of the film with ulvan solution in HPLC grade water was not possible and multilamellar vesicles could not be obtained, with the presence of the polyelectrolyte preventing the phospholipids’ self-assembly in vesicles. On the other hand, when PBS was used as hydration medium, DPPC:ulvan liposomes (LDPPC-UL) were efficiently formed. Their size was around 166 nm and their ζ-potential slightly negative (−5.9 mV) (Table 2). The PDI value and the size distribution curve indicated polydisperse population of particles, probably due to the partial incorporation of ulvan into the DPPC lipid bilayer.

Plain charged liposomes (LDPPG and LDOTAP) exhibited negative and positive ζ-potential values of −34.9 and +60.2 mV, respectively, as expected, according to the charge of the lipids used (Table 2). In addition, both ulvan–containing liposomes exhibited either a negative or positive ζ-potential value according to the employed lipid’s charge. Specifically, under the conditions employed, ulvan–containing liposomes incorporating charged lipids in HPLC grade water were obtained, sizing less than 200 nm, although larger than the respective plain liposomes. The LDPPG-UL liposomes were smaller than 100 nm, with narrow size distribution and, therefore, could be characterized as Small Unilamellar Vesicles (SUVs). Their ζ-potential value was found to be negative (−23.8 mV) [56], but lower than that of the plain charged liposomes. The observed ζ-potential value decrease could be attributed to the localization of ulvan on the surface of the liposomes, hence, decreasing the overall liposomal ζ-potential value due to its lower charge (−15.7 mV). On the other hand, the LDOTAP-UL liposomes’ size was about 175 nm with high nanoparticles’ homogeneity and extremely high positive ζ-potential (+66.2 mV). Therefore, more than a two-fold size increase was observed upon addition of ulvan (from 69.8 to 174.7 nm). It should be noted that there is a strong linear relationship between the ζ-potential and the mole percentage of the charged lipids within a liposome [57]. This means that the change of the molar ratio between the zwitterionic and charged lipids would cause alterations of the ζ-potential values because ζ-potential is strongly dependent on the charge of the lipids. Hence, the ζ-potential values are primarily determined by the charge of the lipid and less affected by the presence of ulvan. Actually, the interactions of ulvan with the lipid membranes also did not significantly differentiate the surface charge of the prepared charged liposomes. The observed size increment and the marginal increase of the surface charge of the ulvan–containing liposomes point to the incorporation of ulvan in the lipid bilayer, in accordance with the DSC results.

The effect of the dispersion medium on the physicochemical characteristics of the liposomes varied. Specifically, the size, PDI value and ζ-potential for LDPPG-UL remained unaffected in PBS. On the contrary, for LDOTAP-UL, an intense increase of the hydrodynamic radius (from 175 nm in HPLC grade water to 289 nm in PBS) and the PDI values was observed (Table 2), while ζ-potential shifted to a less positive value (from +66.2 mV in HPLC grade water to +35.6 mV in PBS). The last observation is quite interesting because, according to the literature, the ζ-potential of DOTAP–containing liposomes is not significantly affected by pH changes, since DOTAP has a quaternary amine at pH values between 3.8 and 7.7 [57]. Therefore, this change of ζ-potential could be attributed to the different conformation of ulvan in PBS and the concealment of the charges in this dispersion medium.

**Figure 3:**
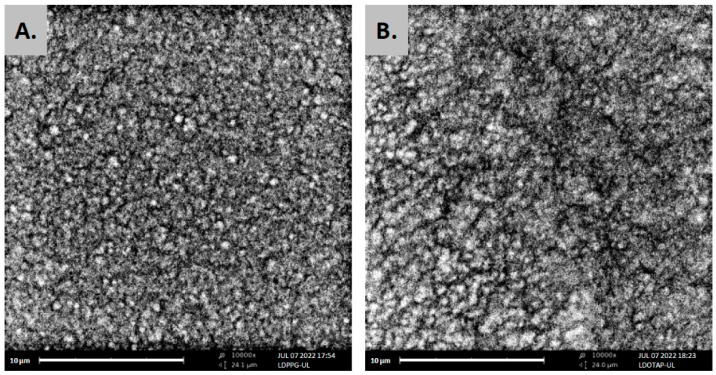
SEM images of ulvan–containing liposomes prepared in HPLC grade water, (**A**) LDPPG-UL, and (**B**) LDOTAP-UL.

**Figure 4:**
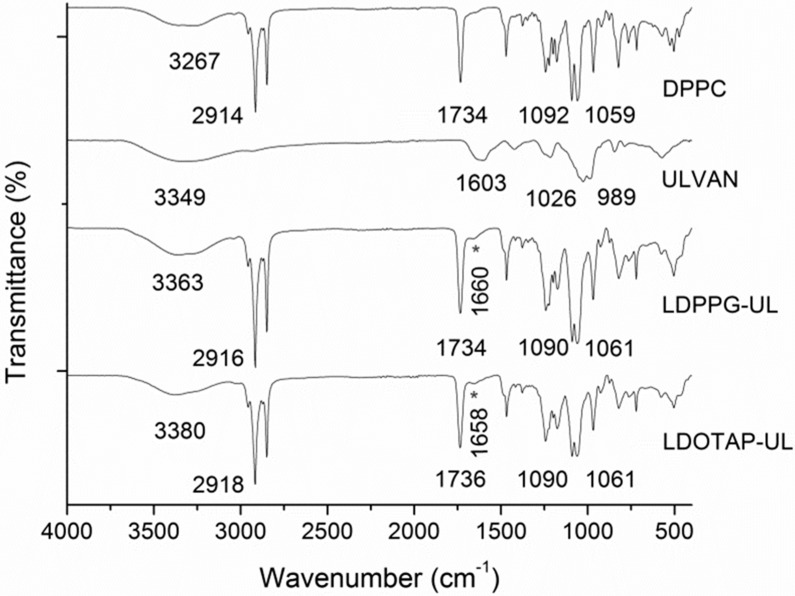
FTIR spectra of DPPC lipid, ulvan, and the ulvan–containing liposomes LDPPG-UL and LDOTAP-UL.

The formation of uniform spherical structures was confirmed by SEM analyses (Figure 3). The FT-IR spectra of ulvan, DPPC, LDPPG-UL, and LDOTAP-UL were obtained to ensure the presence of ulvan in the liposomal preparations (Figure 4). Actually, all characteristic peaks of the lipids were present in the liposomal formulations, along with characteristic absorption bands at 3357 and 1672 cm^−1^ for LDPPG-UL and 3380 and 1658 cm^−1^ for LDOTAP-UL, attributed to the intense characteristic absorption bands of ulvan at 3349 cm^−1^ and 1603 cm^−1^ and assigned to the stretching vibrations of –OH and –C=O carboxyl groups, respectively. The significant shift observed for both absorption bands could be attributed to the conformational changes in ulvan and its interaction with the lipid bilayer.

**Figure 5:**
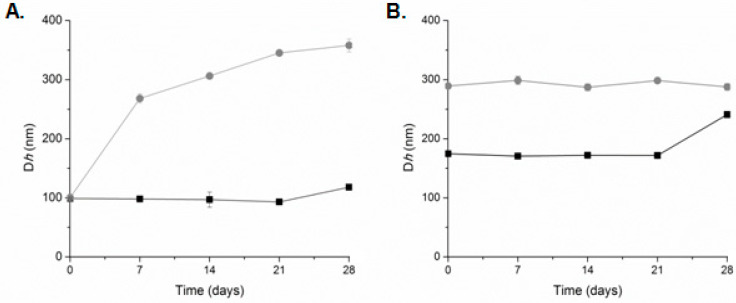
Stability assessment of (**A**) LDPPG-UL and (**B**) LDOTAP-UL liposomes in HPLC grade water (squares, black line) and PBS (circles, grey line) (mean of three independent experiments run in triplicate).

The stability of the prepared systems over time at 4 °C was also assessed (Figure 5). The plain liposomes were found to be stable for at least three months after their preparation [53]. The high ζ-potential values (absolute values) were responsible for the kinetic stability of these systems [56]. When both systems (LDPPG-UL and LDOTAP-UL) self-assembled into HPLC grade water, their physicochemical characteristics (size/D*h*) remained unaffected for 21 days. The PDI and the scattering intensity (data not shown) did not present any significant difference during the stability assessment. After this time period, we observed a small increase of the D*h* values. On the other hand, LDPPG-UL in PBS increased their size from 100 nm (0 day) to 250 nm (7 days) and up to 350 nm (28 days) (Figure 5A). The population of the liposomes became more polydisperse and the scattering intensity values slightly decreased (data not shown). This observation points to spatial differences of ulvan organization into the lipid bilayer, resulting in changes in the physicochemical characteristics of the prepared systems. For LDOTAP-UL, a stable suspension/dispersion was obtained in PBS (Figure 5B). The high values of ζ-potential play a key role in avoiding the aggregation process.

We should also point out that the reconstitution of liposomal cake after lyophilization was more easily achieved in the presence of ulvan (data not shown). At the concentration used, ulvan, as a sulfated polysaccharide, could act as a cryo- and lyo-protectant and stabilize the structure of the liposomes after the reconstitution. It is generally acknowledged that the presence of sucrose, a commonly used cryo- and lyo-protectant, influences the stability of the lipid vesicles, since the sucrose molecules are located on the membrane surface and influence the hydration network by partly replacing the bound water [58]. The stability observed for the ulvan–containing liposomes is also probably associated with the location of the polysaccharide on the lipid membrane.

### 3.3. Preparation and Characterization of Fusidic Acid-Loaded Liposomes

Taking into account the increased physicochemical stability of ulvan–containing liposomes in HPLC grade water, the latter was selected as a suitable medium for drug loading capacity determination and antimicrobial activity testing. For comparison reasons, plain (without ulvan) and ulvan–containing liposomes were loaded with the antibacterial model drug FA using the thin-film hydration method. Both FA-LDPPG and FA-LDOTAP liposomal formulations exhibited sizes of approximately 70 nm with PDI around 0.5 (Table 2). In both cases, the ζ-potential values were in line with the empty liposomes, indicating that the incorporated FA into the lipid bilayers did not alter the surface charge of the lipids (Table 2). On the other hand, FA-LDPPG-UL exhibited more or less the same size as that of FA-LDPPG, whereas in FA-LDOTAP-UL an increase in the size of around 120 nm was observed upon ulvan addition (from 72.1 to 188.6 nm). This increase in size is analogous to the one observed for the empty liposomes (LDOTAP and LDOTAP-UL), and therefore can be exclusively attributed to the presence of ulvan.

**Figure 6:**
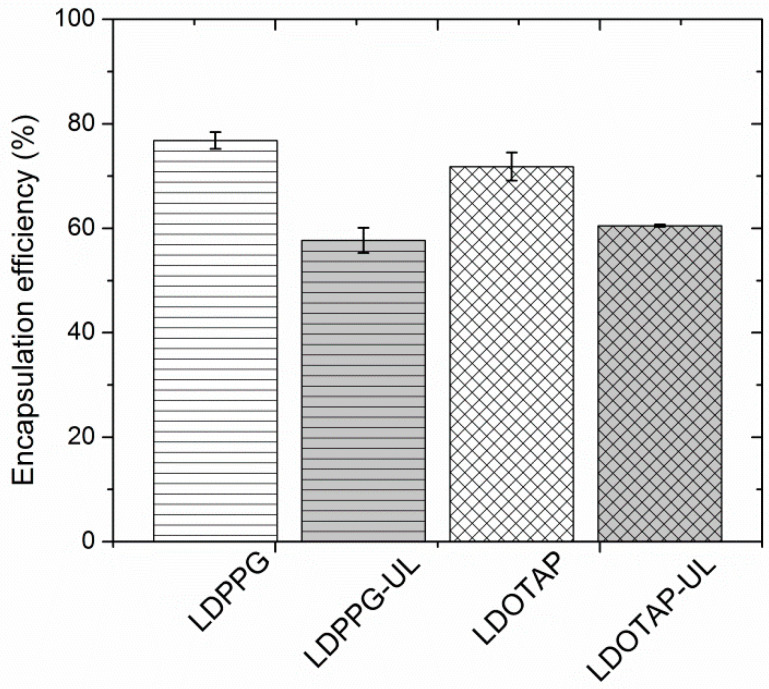
Fusidic acid (FA) entrapment efficiency (%) of plain (LDPPG, LDOTAP) and ulvan–containing (LDPPG-UL, LDOTAP-UL) liposomes.

In Figure 6 the entrapment efficiency of the different liposomal formulations, expressed as % *w/w*, is depicted. Entrapment efficiency of the different formulations was found to range between 57.7 and 75.7%. Due to the lipophilic nature of FA (log P = 4.42), the developed systems were able to offer higher than 55% of drug entrapment, with the highest value observed for FA-LDPPG liposomes. Such FA entrapment efficiencies have been previously described for DPPC:Chol:Span 80 liposomes [50], ascribed to the presence of an optimum concentration of surfactant, which eventually increases the solubility of drug in the lipid layers.

The plain liposomes entrapped the highest amount of FA (70 to 76%), which is significantly higher than that calculated for the ulvan–containing formulations (58 to 60%) (Figure 6). Both ulvan–containing liposomes were characterized by an entrapment efficiency that did not significantly differ from one another, indicating that the presence of ulvan, rather than the type of lipids used, mainly affects the entrapment efficacy [25]. In our case, the ulvan embedded in the liposomal structure may disorganize the structure of the lipid bilayers and provide less space for FA inside the lipidic compartments of the ulvan–containing liposomes [59].

### 3.4. Evaluation of the Antibacterial Activity of the Liposome Nanocarriers

FA is an established anti-staphylococcal agent that has been used in clinical practice for more than four decades. Previous studies have demonstrated that susceptibility of staphylococci to FA is observed at MICs of ≤1 μg/mL and resistance at MICs of ≥4 μg/mL [60].

The MIC values of the FA-loaded liposomes, along with that of pure FA against *S. aureus* were determined (Table 3). It was observed that all formulations, regardless of the type of lipid used and the presence or absence of ulvan, inhibited the growth of *S. aureus* at similar concentrations (C_LIPOSOMES_ 5.0–6.3 μg/mL). Nevertheless, when MIC values were expressed as the concentration of FA contained in each liposomal formulation (MIC_FA_), calculated on the basis of the entrapment efficiency observed for each liposome, noteworthy differences were observed according to the liposomal charge. Specifically, the negatively charged FA-loaded liposomes, both plain and ulvan–containing (FA-LDPPG and FA-LDPPG-UL, respectively), presented inhibitory activity at an FA concentration of 0.2 μg/mL, equal to that of pure FA. On the other hand, both positively charged FA-loaded liposomes (FA-LDOTAP and FA-LDOTAP-UL) exhibited lower MIC_FA_ values (0.1 μg/mL). The lower MIC_FA_ values of the DOTAP–containing liposomes could be associated with their positive charge, triggering interactions with the negatively charged bacteria and promoting fusion [61]. The ζ-potential of *S. aureus* has been reported to be −30.5 mV, since its cell membrane contains a teichoic acid layer which contributes to the overall negative ζ-potential value [62,63].

In the employed liposomal concentrations, ulvan concentration was much lower (theoretically estimated as ~0.2 μg/mL) than the one found to inhibit bacterial growth (156 μg/mL). Therefore, our results suggest that the presence of lipids that favour interactions between liposomes and bacterial cells is the key factor for enhanced drug delivery.

Previous work on ciprofloxacin-loaded liposomes has shown that fusion of liposomes with bacterial cell walls resulted in the direct release of ciprofloxacin inside the bacterial cells (the site of action) [64,65]. Furthermore, studies on microbial susceptibility suggested that fusion mechanism is beneficial in enhancing antibacterial activity of ciprofloxacin [63]. Analogously, the delivery of the oil-soluble bactericide Triclosan was studied for a number of charged liposomal compositions. Superior delivery of the liposomal bactericide relative to the free bactericide occurred for positively charged liposomes (DPPC:Chol:stearylamine), while targeted delivery to *Staphylococcus epidermidis* was observed at low bactericide concentrations by negatively charged liposomes (DPPC:DPPG and DPPC:phosphatidylinositol) [66,67].

## 4. Conclusions

In preformulation studies, we demonstrated the interactions developed between the marine sulfated polysaccharide ulvan and neutral or charged phospholipids by employing DSC. Depending on the charge of the lipid membranes employed, the presence of ulvan both on the surface and inside the lipid bilayer was evidenced. This is most probably due to the well-known amphiphilic nature of ulvan, imposing changes in its spatial conformation according to the lipidic environment. The results from the DSC studies served as a roadmap for the design and development of novel ulvan–containing liposomes, loaded or not with the model antibacterial hydrophobic drug fusidic acid. Hence, ulvan–containing charged liposomes readily occurred upon addition of ulvan solutions to lipid films containing either negatively or positively charged phospholipids. By analogy to the DSC results, the size and surface charge analysis of the resulting liposomes implied the surface interactions or incorporation of ulvan within the negatively or positively charged lipid bilayer, respectively. Specifically, the developed liposomes sized in the nanometer scale were of highly charged surface and facilitated the entrapment of fusidic acid in sufficient amounts that exhibited equal or better antibacterial activity in vitro against *S. aureus* in comparison to pure fusidic acid.

## Figures and Tables

**Table 1:** Calorimetric profiles of lipid:ulvan bilayer systems (upon heating).

System	C_ulvan_ (mg/mL)	T_onset.m_ (°C) ^a^	T_m_ (°C) ^b^	ΔT_1/2.m_ (°C) ^c^	Δ*H*_m_ (J/mol) ^d^	T_onset.s_ (°C) ^a^	T_s_ (°C) ^b^	ΔT_1/2.s_ (°C) ^c^	Δ*H*_s_ (J/mol) ^d^
DPPC: ulvan	0	40.9	41.9	1.33	−398.0	34.2	36.5	2.08	−36.6
DPPC: ulvan	0.5	40.8	41.7	1.29	−378.0	33.9	36.2	1.98	−38.9
DPPC: ulvan	1	41.2	42.5	1.84	−226.1	36.0	37.5	1.83	−37.4
DPPC: ulvan	2.5	41.2	42.5	1.84	−371.0	36.0	37.5	1.93	−16.2
DPPC: ulvan	5	41.4	43.0	1.89	−371.0	33.8	37.8	1.55	−8.9
DPPC: ulvan	7.5	41.3	42.9	2.02	−362.0	32.2	34.5	5.20	−2.7
DPPC: ulvan	10	41.2	43.3	1.91	−398.3	36.8	37.4	1.79	−2.6
DPPC:DPPG: ulvan	0	42.0	43.1	0.74	−287.4	34.9	36.9	1.19	−41.2
DPPC:DPPG: ulvan	0.5	41.9	42.9	0.76	−276.8	-	-	-	-
DPPC:DPPG: ulvan	1	40.1	41.9	1.37	−98.5	-	-	-	-
DPPC:DPPG: ulvan	2.5	41.0	42.3	1.37	−92.0	-	-	-	-
DPPC:DPPG: ulvan	5	41.0	42.5	1.67	−164.2	-	-	-	-
DPPC:DPPG: ulvan	7.5	41.8	42.8	1.11	−43.4	-	-	-	-
DPPC:DPPG: ulvan	10	41.2	42.8	3.47	−122.8	-	-	-	-
DPPC:DOTAP: ulvan	0	33.2	36.6	3.25	−398.3	-	-	-	-
DPPC:DOTAP: ulvan	0.5	33.4	36.9	3.48	−402.3	-	-	-	-
DPPC:DOTAP: ulvan	1	33.8	37.1	3.50	−414.2	-	-	-	-
DPPC:DOTAP: ulvan	2.5	34.1	37.1	3.62	−449.8	-	-	-	-
DPPC:DOTAP: ulvan	5	34.8	37.0	3.15	−464.4	-	-	-	-
DPPC:DOTAP: ulvan	7.5	34.9	37.5	3.40	−477.5	-	-	-	-
DPPC:DOTAP: ulvan	10	34.4	37.3	3.40	−494.5	-	-	-	-

^a^ T_onset_: temperature at which the thermal event starts; ^b^ T: temperature at which heat capacity (Δ*C_p_*) at constant pressure is maximum; ^c^ ΔT_1/2_: half width at half peak height of the transition; ^d^ Δ*H*: transition enthalpy normalized per mol of lipid bilayer system. m: main transition; s: secondary transition.

**Table 2:** The physicochemical characteristics of pure ulvan (UL) and plain (LDPPC, LDPPG, LDOTAP), ulvan–containing (LDPPC-UL, LDPPG-UL, LDOTAP-UL), and FA-loaded (FA-LDPPG, FA-LDOTAP, FA-LDPPG-UL, FA-LDOTAP-UL) liposomal formulations.

System	Dispersion Medium	C_ulvan_ (mg/mL)	D*h* (nm) ^a^	PDI ^b^	ζ-Potential (mV)
UL	HPLC grade water	1	578.8 ± 76.0	1.000 ± 0.000	−15.7 ± 1.6
LDPPC	HPLC grade water	-	123.3 ± 15.8	0.652 ± 0.053	6.5 ± 1.9
LDPPG	HPLC grade water	-	73.8 ± 7.9	0.362 ± 0.007	−34.9 ± 4.3
LDOTAP	HPLC grade water	-	69.8 ± 5.9	0.318 ± 0.005	60.2 ± 2.8
LDPPG-UL	HPLC grade water	1	98.9 ± 1.5	0.443 ± 0.002	−23.8 ± 4.5
LDOTAP-UL	HPLC grade water	1	174.7 ± 1.8	0.393 ± 0.030	66.2 ± 4.0
UL	PBS	1	432.9 ± 17.0	1.000 ± 0.000	−10.1 ± 0.1
DPPC-UL	PBS	1	165.9 ± 2.3	0.752 ± 0.022	−5.9 ± 1.6
LDPPG-UL	PBS	1	100.4 ± 1.9	0.428 ± 0.009	−25.6 ± 0.8
LDOTAP-UL	PBS	1	289.2 ± 0.9	0.482 ± 0.081	35.6 ± 1.9
FA-LDPPG	HPLC grade water	-	71.0 ± 3.5	0.572 ± 0.014	−32.1 ± 0.6
FA-DOTAP	HPLC grade water	-	72.1 ± 1.4	0.563 ± 0.009	62.9 ± 5.4
FA-LDPPG-UL	HPLC grade water	1	65.2 ± 4.1	0.512 ± 0.011	−20.0 ± 1.8
FA-LDOTAP-UL	HPLC grade water	1	188.6 ± 2.7	0.507 ± 0.015	54.3 ± 1.7

^a^ Hydrodynamic diameter; ^b^ Polydispersity Index.

**Table 3:** Antibacterial activity (expressed as MIC values) of the FA-loaded liposomal formulations (FA-LDPPG, FA-LDPPG-UL, FA-LDOTAP, and FA-LDOTAP-UL) and of pure fusidic acid (FA) solution used as control.

	C_LIPOSOMES_ (μg/mL) ^a^	MIC_FA_ (μg/mL) ^b^
FA-LDPPG	6.3	0.2
FA-LDPPG-UL	5.2	0.2
FA-LDOTAP	5.0	0.1
FA-LDOTAP-UL	6.2	0.1
FA	-	0.2

^a^ Concentration of the respective FA-loaded liposomes at which growth inhibition of *S. aureus* is observed; ^b^ Concentration of FA in the liposomes, as calculated on the basis of the entrapment efficiency determined for each liposome, inhibiting the growth of *S. aureus.*

## Data Availability

The data that support the findings of this study are available from the corresponding authors upon request.
